# The Mechanism of Excessive Intestinal Inflammation in Necrotizing Enterocolitis: An Immature Innate Immune Response

**DOI:** 10.1371/journal.pone.0017776

**Published:** 2011-03-21

**Authors:** Nanda Nanthakumar, Di Meng, Allan M. Goldstein, Weishu Zhu, Lei Lu, Ricardo Uauy, Adolfo Llanos, Erika C. Claud, W. Allan Walker

**Affiliations:** 1 Developmental Gastroenterology Laboratory, Harvard Medical School, Boston, Massachusetts, United States of America; 2 Pediatric Surgical Research Laboratory, MassGeneral Hospital for Children, Harvard Medical School, Boston, Massachusetts, United States of America; 3 Institute of Nutrition and Food Technology, University of Chile, Santiago, Chile; 4 Section of Neonatology, Department of Pediatrics and Medicine, The University of Chicago, Chicago, Illinois, United States of America; University of Florida, United States of America

## Abstract

Necrotizing enterocolitis (NEC) is a devastating neonatal intestinal inflammatory disease, occurring primarily in premature infants, causing significant morbidity and mortality. The pathogenesis of NEC is associated with an excessive inflammatory IL-8 response. In this study, we hypothesized that this excessive inflammatory response is related to an immature expression of innate immune response genes. To address this hypothesis, intestinal RNA expression analysis of innate immune response genes was performed after laser capture microdissection of resected ileal epithelium from fetuses, NEC patients and children and confirmed in *ex vivo* human intestinal xenografts. Changes in mRNA levels of toll-like receptors (TLR)-2 and -4, their signaling molecules and transcription factors (MyD88, TRAF-6 and NFκB1) and negative regulators (SIGIRR, IRAK-M, A-20 and TOLLIP) and the effector IL-8 were characterized by qRT-PCR. The expression of TLR2, TLR4, MyD88, TRAF-6, NFκB1 and IL-8 mRNA was increased while SIGIRR, IRAK-M, A-20 and TOLLIP mRNA were decreased in fetal vs. mature human enterocytes and further altered in NEC enterocytes. Similar changes in mRNA expression were observed in immature, but not mature, human intestinal xenografts. Confirmation of gene expression was also validated with selective protein measurements and with suggested evidence that immature TRL4 enterocyte surface expression was internalized in mature enterocytes. Cortisone, an intestinal maturation factor, treatment corrected the mRNA differences only in the immature intestinal xenograft. Using specific siRNA to attenuate expression of primary fetal enterocyte cultures, both TOLLIP and A-20 were confirmed to be important when knocked down by exhibiting the same excessive inflammatory response seen in the NEC intestine. We conclude that the excessive inflammatory response of the immature intestine, a hallmark of NEC, is due to a developmental immaturity in innate immune response genes.

## Introduction

Necrotizing enterocolitis (NEC) is the most common gastrointestinal emergency in premature infants, affecting almost ten percent of all infants with a birth weight under 1500 g [1], [2]. Up to forty percent of afflicted premature infants require intestinal resection with a mortality rate of almost fifty percent and significant subsequent morbidity (e.g., short bowel syndrome, etc) [2], [3] The cost in medical care annually is in excess of five hundred million dollars [2], [3], [4]. Bacterial colonization is required for the initial inflammatory condition that precedes overt NEC [2], [3], [5]. The additional factors that allow colonization-induced inflammation to progress to fulminant, life-threatening forms of NEC remain elusive, although an immature intestine represents the most important risk factor. Furthermore, NEC can be prevented by glucocorticoids, an intestinal maturation factor, if given prenatally [2], [5], [6]. Thus, defining the pathophysiologic basis of NEC and devising strategies for its prevention is a top priority in neonatal gastrointestinal medicine.

This laboratory has had a longstanding interest in the development of host defense in the human intestine and the extent to which immaturities in host defense influence the expression of age-related neonatal gastrointestinal disease states such as NEC. As stated above, the most important risk factor for this condition as reported from several large studies, is prematurity [4], [5]. Accordingly, using established intestinal models for human gut development [7]–[9], we have systematically examined the interaction of colonizing bacteria with the developing human gut. We have previously reported that the immature gut reacts to molecular patterns on colonizing bacteria and to endogenous inflammatory stimuli by mounting an excessive inflammatory (IL-8) response [8], [9].

Recently, we have reported that the excessive inflammatory response of the immature intestine is in part due to a developmental underexpression of IκB, an important regulator of NFκB signaling [10] and that the excessive inflammatory response could be dampened with hydrocortisone, a known maturational factor that regulates intestinal development [9]. Therefore, we have hypothesized that a more generalized immaturity of the intestinal innate immune response may contribute to the observed excessive inflammation in the immature intestine and thus in part be involved in the onset of NEC. Accordingly, in this study we compared the epithelial gene expression and selective protein levels of the innate immune inflammatory response in fetal and older child enterocytes and in selective primary enterocytes from freshly resected NEC patients. Cellular observations were then confirmed in *ex vivo* fetal intestinal xenografts. The results of these observations suggest that multiple genes involved in the innate immune response may be developmentally regulated.

## Results

**Figure 1:**
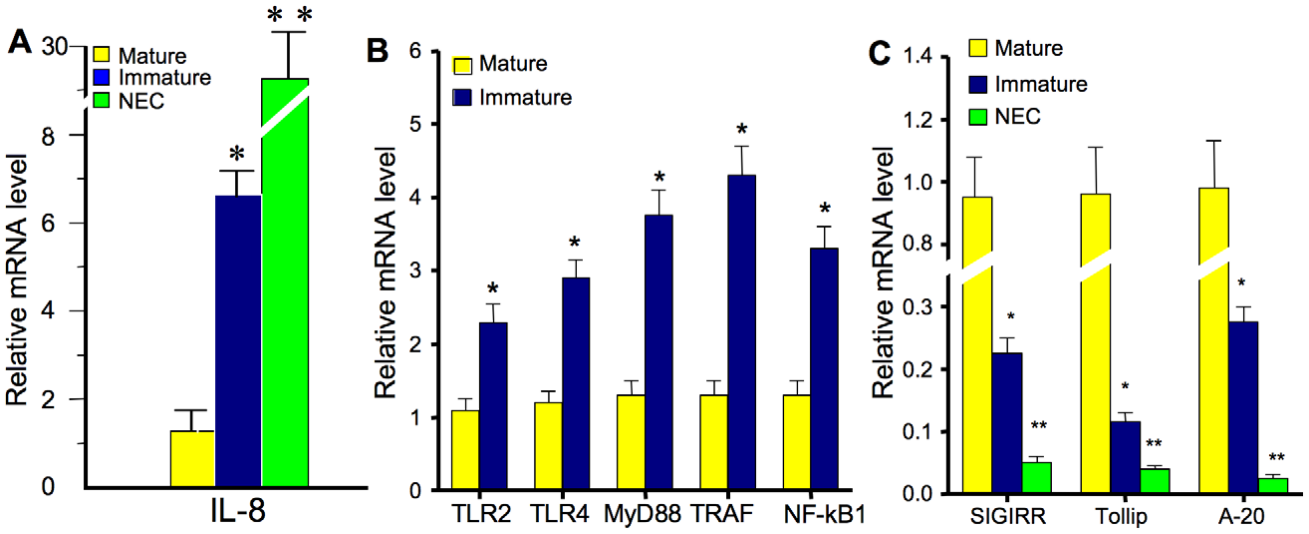
NFκB/MyD88 acute innate immune inflammatory and negative response genes as measured by real time PCR and expressed by relative fold mRNA levels of laser capture epithelial microdissection of three fetal/NEC/control intestines with control mRNA levels arbitrarily expressed as one (A) IL-8 response; (B) receptors/signaling molecules/transcription factor (fetal/control intestine only); and (C) negative regulators (*p<0.01; ** p<0.001).

NFκB/MyD88 innate inflammatory genes are over-expressed and negative regulator genes are underexpressed in fetal and NEC intestine. Using laser capture microdissection (LCM) of ileal resections from 1) three children subjected to elective intestinal surgery, 2) three therapeutically aborted fetus and 3) three NEC patients, epithelial RNA was isolated and innate immune response genes quantitated by qRT-PCR. Due to a scarcity of epithelial RNA from NEC resections, mRNA levels are only shown in Figures 1A and 1C. Figure 1A shows a highly significant increase in enterocyte-related IL-8 mRNA in fetal (more so in NEC) vs. child intestine. Figure 1B depicts the fold difference relative to mature intestine for TLR2 and TLR4, MyD88, TRAF6, NF-κB1 and IL-8 mRNA. Fold differences are comparison of mRNA levels in genes from older children, arbitrarily set at one-fold, with levels determined in fetal epithelial RNA. Previous studies have also shown that mature enterocytes regulate the innate immune inflammatory response by expressing specific negative regulators of innate inflammatory response genes which result in controlled activation to inflammatory stimuli [11]–[13]. Several negative regulators have been shown to be expressed in the mature enterocyte [13], [14]. Therefore, using epithelial RNA isolated by LCM from children, fetuses and NEC resected intestine and qRT-PCR analysis, we also determined mRNA levels for SIGIRR (a cell surface negative regulator), TOLLIP and A-20 (intracellular negative regulators) as representative epithelial regulators. As stated above, the differences depicted in **Figure 1C** are a comparison of mRNA negative regulator genes from older children, arbitrarily set at one-fold, with levels determined in both fetal and NEC epithelial RNA. SIGIRR, TOLLIP and A-20 mRNA was reduced by 80%, 90% and 70% respectively in fetal vs. older child intestine and further decreased by approximately 95% in NEC vs. older child intestine. These observations suggest that the excessive intestinal inflammatory response to stimulation in immature intestine may be due to the developmental overexpression of epithelial NFκB/MyD88 innate immune response genes and the underexpression of negative regulators. An associated observation is that the epithelium in NEC may show additional altered developmental expression.

### Innate immune response and negative regulator genes are developmentally regulated in immature fetal intestinal xenografts

**Figure 2:**
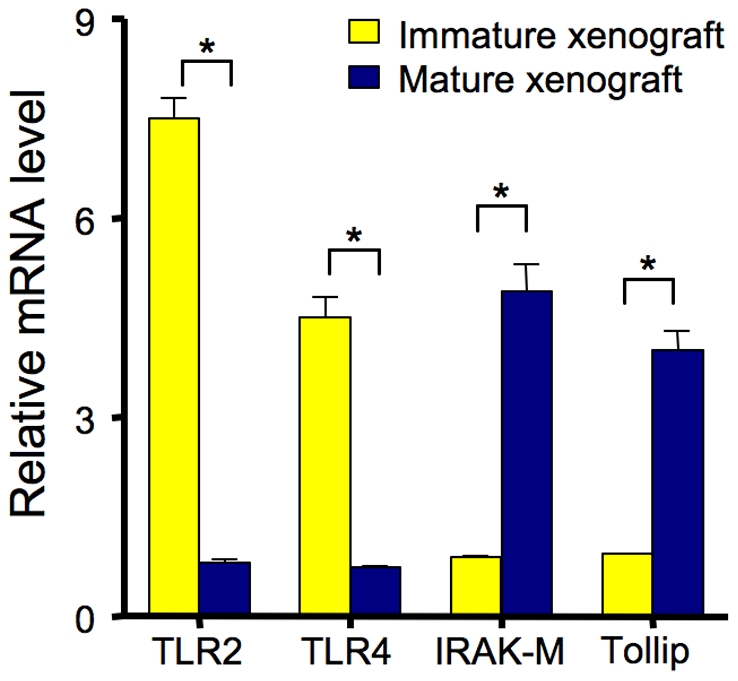
Confirmation of the observations in **Figure 1** in immature/mature xenografts as measured by relative mRNA expression level of laser capture epithelial microdissections (*p<0.01) with mature vs. immature mRNA fold levels (mature) arbitrarily set at one.

In previous publications using fetal intestinal xenografts [7]–[9], [15], we have been required to confirm isolated cellular observations in selected samples by repeating the observation in fetal intestinal xenografts, thought to be a more physiologically relevant *ex vivo* model. In these studies, we have shown a striking difference in the inflammatory (IL-8) response to exogenous and endogenous stimuli (LPS and IL-1β) between xenografts at 20 wks posttransplant (designated immature xenografts) compared to xenografts at 30 wks posttransplant (designated mature xenografts) [7], [8]. Therefore, we determined the mRNA of innate immune response genes in this model. Accordingly, using LCM of the epithelium RNA of five immature/mature xenografts, we determined TLR-2 and 4, IRAK-M and TOLLIP mRNA. **Figure 2** shows a striking increased in TLR-2 and 4 and decrease in IRAK-M/TOLLIP in immature vs. mature xenografts to confirm the observations that these genes are developmentally regulated. Again the fold mRNA comparison between the immature and mature intestine was determine by arbitrarily assigning in mRNA of TLR2 and 4 a value of one-fold for a mature intestine and for IRAK-M and TOLLIP assigning an arbitrary one-fold value for immature intestine. A significant (p<0.01) difference was noted between the two in each instance.

### Glucocorticoids accelerate development of innate inflammatory gene expression in immature but not mature intestinal xenografts

**Figure 3:**
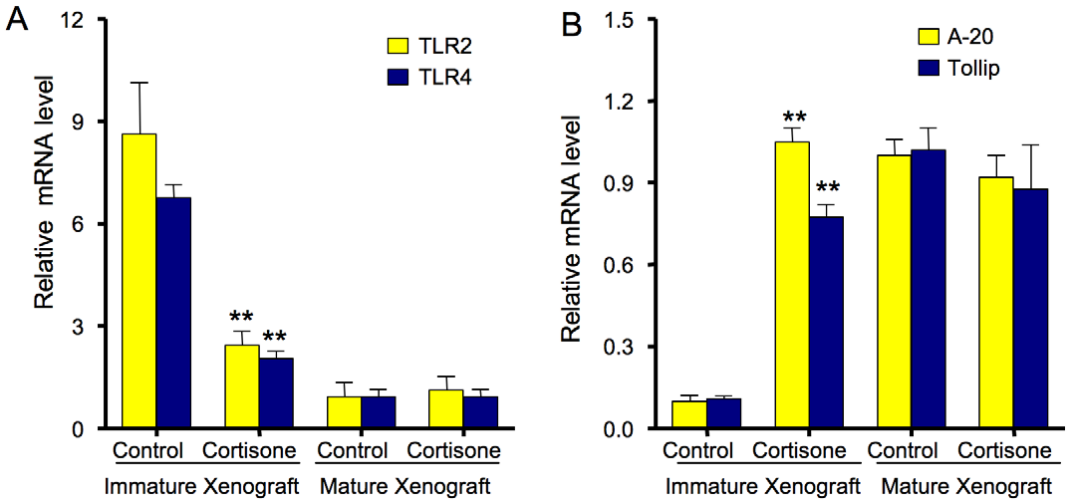
The effect of cortisone acetate treatment on TLR2/4 and A-20/TOLLIP genes in immature and mature xenografts. NOTE: The reduced response occurred only in immature xenografts (**p<0.001).

We chose to use the *ex* vivo xenograft model to demonstrate the developmental regulation of innate immune response genes for several reasons. 1) The time needed to expose enterocytes to hydrocortisone (5 days) precluded using primary enterocytes particularly from NEC patients; 2) we have previously reported that hydrocortisone injection into SCID mice followed by an inflammatory stimuli (IL-1β) reduced the IL-8 response only in immature xenografts and qRT-PCR measurement of IκB mRNA from LCM of epithelial RNA one week after steroid treatment resulted in an increased IκB gene expression [9]. Therefore, we reasoned that using an established and partially characterized model would represent the best approach to showing a developmental basis of the immature innate immune response. Accordingly, to further confirm the cellular observations in an *ex vivo* model of human intestinal development, we determined the effect of cortisone on the expression of selective innate inflammatory response genes in human intestinal xenografts. Both immature (20-week-old) and mature (30-week-old) human intestinal xenografts were treated with cortisone acetate or a vehicle control, as described previously [9]. After one week, total RNA from intestinal xenografts was isolated and mRNA levels of TLR2, TLR4, A-20 and TOLLIP were determined by qRT-PCR (**Figure 3**). As before, the level of mRNA from control xenografts was arbitrarily set and then compared to xenografts after corticosteroid treatment.TLR2 and TLR4 mRNA fold levels relative to controls were reduced significantly (p<0.001) by cortisone treatment in immature xenografts but no significant difference was observed in mature xenografts (p>0.4) (**Figure 3A**). In contrast, cortisone treatment significantly induced A-20 and TOLLIP mRNA fold levels relative to controls (p<0.001), only in immature but not mature xenografts (**Figure 3B**). These data suggested that the difference in innate inflammatory response genes in enterocytes isolated from immature vs. mature human intestinal xenografts is in part due to a developmental regulation of genes that affect the innate immune response including negative regulation.

### IL-8 and TLR-2 mRNA levels are higher and A-20/TOLLIP mRNA and protein levels are lower in fetal vs. NEC primary enterocytes

**Figure 4:**
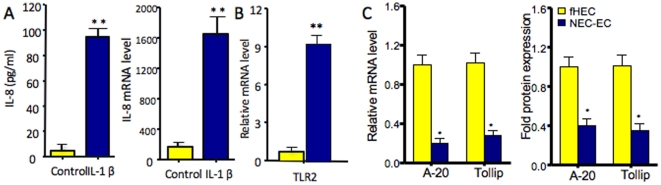
IL-8, TLR2, A-20/TOLLIP mRNA and protein expression in fetal vs. NEC primary enterocytes. Fold difference in IL-8 mRNA and secreted protein levels after IL-1β (10 µg) stimulation **(** **Figure 4A** ; **p<0.001), TLR2 mRNA **(** **Figure 4B** ; **p<0.001) and A-20/TOLLIP mRNA (*p<0.01) and protein (*p<0.01) **(** **Figure 4C** **)** were noted when fetal vs. NEC primary cells were compared.

To further compare the functional difference in the effector, TLR and negative regulators in primary enterocytes isolated from fetal and NEC ileum, we determined the fold difference in mRNA and protein. **Figure 4A–C** depicts the results of the primary cell isolations from fetal and NEC enterocytes. When stimulated with IL-1β (10 µg), IL-8 mRNA and protein from NEC cells was significantly higher than fetal cells (p<0.001) (**Figure 4A**). TLR-2 mRNA was also significantly greater in NEC than fetal cells (p<0.001) (**Figure 4B**) In contrast, A-20/TOLLIP mRNA and protein were significantly reduced in NEC vs. fetal cells (p<0.01). These experiments, unlike corticosteroid studies, can be accomplished in primary enterocytes from fetal and NEC resections with a finite period of viability because of the shortened time of experimentation. In addition, as with previous experiments fold differences between fetal and NEC cells were compared by assigning an arbitrary fold value of one to fetal cells before comparing with NEC cells. These results support previous observations and provide a functional relevance for underexpressed negative regulators in the excessive inflammatory response in NEC.

### Role of negative regulators in the inflammatory response of primary fetal enterocyte cultures

**Figure 5:**
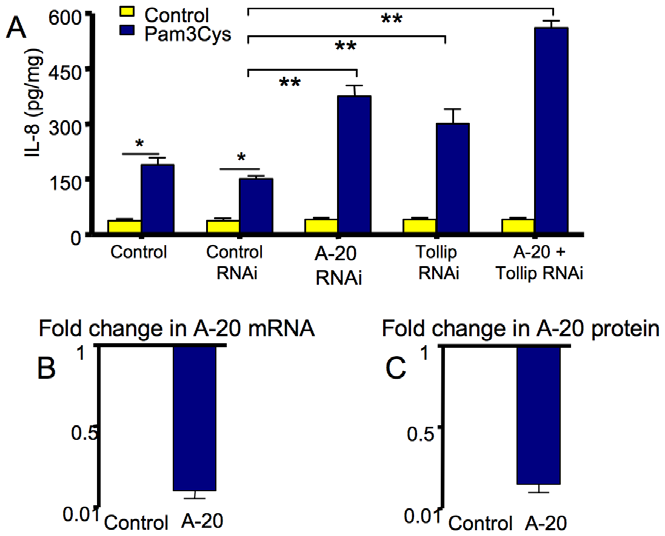
RNAi knockdown of A-20 and TOLLIP in fetal enterocytes followed by PAM3Cys (TLR2 ligand) stimulus. (**A**) The IL-8 cytokine level after knockdown increased to that shown in NEC enterocytes. The representative efficiency of (**B**) A-20 knockdown of mRNA and (**C**) protein was appropriate for the altered observation as compared to a control level of one. (*p<0.01; ** p<0.001).

To determine if the expression of negative regulators was in part responsible for the highly significant lower fold IL-8 mRNA and protein expression in response to inflammatory stimuli in fetal vs. NEC-cells (**Figure 4A**), knockdown experiments in fetal cells were performed with A-20 and TOLLIP specific siRNA prior to treatment with Pam3Cys (10 µg/ml) a TLR-2 ligand. **Figure 5A** depicts a significant increase in IL-8 secretion (p<0.01) after Pam3Cys stimulation. Furthermore, knockdown of each negative regulator led to an enhanced IL-8 induction (p<0.001) and knockdown of both negative regulators enhanced IL-8 induction further in an additive manner. **Figure 5B** show the degree of knockdown of A-20 mRNA and protein respectively using this technique. Similar results were obtained when Pam3Cys-induced IL-6 expression was analyzed (data not shown). As with other studies, an arbitrary fold level of one was chosen for A-20 mRNA and protein in controls compared to post-knockdown. These observations suggest a functional role for negative regulator genes in the immature intestinal inflammatory response and a probable role in NEC.

### Epithelial TLR4 expression in the immature and mature intestine

**Figure 6:**
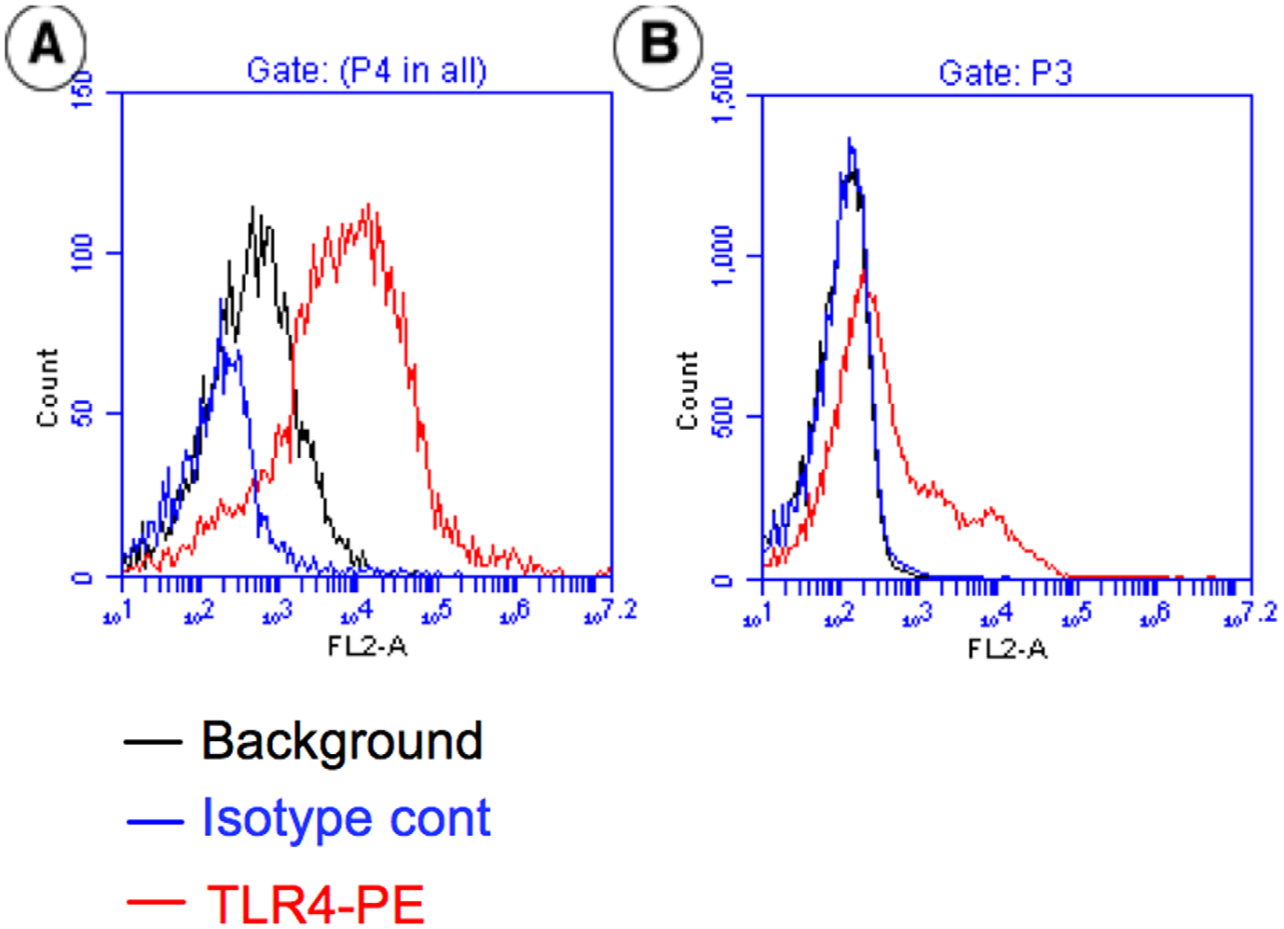
TRL4 expression in immature and mature enterocytes. **A** and **B**: FACS analysis of cell surface expression of TLR4. **A**: fetal enterocyte (H4 cells); **B**: mature enterocyte (Caco-2 cells).

We have previously published an observation to suggest that excessive TLR4 receptors are expressed on the surface of fetal enterocytes (H4) cells and these receptors are further unregulated with inflammation [16]. We also know from other studies using Caco2 cells as an example of mature intestinal enterocytes that TLR4 receptors are internalized [17]. Accordingly, we performed FACS analysis of the enterocyte culture model for immature fetal intestine (H4 cells) and the mature intestine (Caco-2 cells). As observed in the fetal intestine, H4 cells expressed robust TLR4 on the cell surface **(** **Figure 6A** **)** but the surface expression was significantly diminished in Caco-2 cells, an enterocyte model for the mature intestine (**Figure 6B**). These preliminary observations are consistent with the level of the TLR4-mediated IL-8 response in immature and mature cell types reported previously [8].

## Discussion

We have previously reported that human premature enterocytes respond excessively to exogenous and endogenous stimuli to produce an increased inflammatory (IL-8) response [7]–[9]. In addition, this excessive inflammatory response can be reduced to that occurring in the mature enterocyte by the trophic factor hydrocortisone [9]. Recently, we reported that the expression of the IκB gene, a key modulator of inflammatory cytokine transcription by NFκB, was reduced in fetal human enterocytes and preweaned, immature rat intestine [10], suggesting a developmental regulation of innate inflammation in the immature gut. Accordingly in this study, the expression of intestinal innate inflammatory response genes were analyzed by qRT-PCR using epithelial RNA obtained by laser capture microdissesction from fetal and NEC small intestinal resections and surgical specimens from older children. Over expression of Toll-like receptors (TLR2 and TLR4), their intra-cellular signaling molecules (e.g., MyD88, TRAF6, etc.) and a transcriptional factor (NFκB_1_) as well as the effector IL-8 genes was noted in immature fetal intestine compared to intestine from children. Because of the limitation of NEC epithelial RNA, only selective genes were noted to be further overexpressed in the epithelium of freshly resected NEC tissue (**Figure 1A & C**). We also analyzed genes involved in negative regulation of the NFκB signaling response (SIGIRR, IRAK-M, TOLLIP and A-20) and noted that these genes were downregulated, again more so in NEC enterocytes (**Figure 1C**). Therefore, these data suggest that the altered expression of these genes in fetal, and particularly in NEC small intestine, may help explain the initial excessive inflammatory response to colonizing bacteria and their microbial associated molecular pattern molecules (MAMP) that characterizes the premature who develops NEC [**17**].

After birth, the intestinal epithelium, particularly the distal ileum and colon, are suddenly continuously exposed to excessive quantities of MAMPs from initial colonizing bacteria. In term infants, the maturing enterocyte has devised protective mechanisms to prevent chronic inflammation from this exposure. These mechanisms include a reduction in TLR surface expression [13], [16], [18] and post-receptor signal transduction complexes that are required for activation of the NFκB pathway [10], resulting in a reduced transcription of inflammatory chemokines (IL-8, IL-6) and cytokines (TNFα and IL-1β). In addition, an increased expression of surface and intracellular negative regulators to reduce the response of TLR signaling in mature enterocytes, so called TLR tolerance, is also operational to modulate the inflammatory cascade to prevent persistent inflammation [12]–[14], [19]. For example, acquisition of endotoxin tolerance in human intrahepatic biliary epithelium is mediated by induction of the negative regulator, IRAK-M [20]. Although, the ontogenic control of postnatal tolerance is incompletely understood, the developmental changes reported in this study that coordinates the downregulation of cell surface expression of TLRs and their signaling molecules and upregulation of negative regulators is likely to be important in postnatal adaptation to initial intestinal colonization. Accordingly, we hypothesize that when an infant is born prematurely before proper programming of this developmental event, they may inappropriately respond to colonization with excessive inflammation that may lead to NEC. In addition, we have also previously reported [10] that primary fetal enterocyte cultures (H4 cells) respond to non-pathogenic colonizing bacteria with an inflammatory response unlike the models of mature enterocytes (NCM, Caco-2, T84 cells) which do not respond to those colonizing bacteria [8], [10], [16], another cause for excessive inflammation.

TLRs on enterocytes *in utero* are in a relatively germfree environment and therefore are not as vulnerable to microbial inflammatory stimuli as are those in infants in the extrauterine environment. Why are they expressed in abundance and on the enterocyte surface *in utero*? Toll receptors originally reported in *Drosophila* were shown to have developmental functions mediating dorsal/ventral orientation during development [21]. The innate immune response function occurred only after maturation. Although speculative, this same differential function of TLRs from the intrauterine to the extrauterine environment could pertain with humans. This speculation, however, will require further studies to confirm.

Several recent studies in rodent models for NEC have implicated a persistently increased TLR expression on immature enterocytes and excessive NFκB signal transduction as the basis for inflammatory necrosis [17]–[19], [22], [23]. The surface enterocyte expression of TLR4 was necessary for intestinal inflammation in a rat and mouse model for NEC and TLR4 mutant mice were unable to express NEC histopathology [18]. Furthermore, primary enterocytes isolated from newborn mice delivered vaginally had a reduction in TLR4-mediated inflammation in part due to a postpartum decrease in functional IRAK1 kinase, a key signaling molecule of TLR signaling [18]. In contrast, enterocytes from mice delivered by cesarean section had a sustained inflammatory response to LPS because of a persistently high surface TLR4 expression. In addition, increased expression of TLR4 in neonatal rodent enterocytes resulted in a decreased reproliferation of these enterocytes in response to inflammatory necrosis via an inhibition of β-catenin signaling [17], [18]. We have previously reported that a fetal human enterocyte cell line, H4 cells, have an abundance of TLR4 surface receptors which are increased in expression with inflammation [16]. In this study, we have shown by FACS analysis, using anti-TLR4 antibodies in fetal vs. mature enterocyte cell lines (**Figure 6**), that TLR4 receptors are expressed on the surface of fetal enterocytes but in the cytoplasm of mature enterocytes. These data suggest that the reduction of TLR4 surface expression may be an important mechanism for downregulating the innate immune inflammatory enterocyte response to colonizing bacteria in the post partum period. It needs to be emphasized that this observation is preliminary and additional studies are need to confirm our conclusions. Furthermore, the mechanism of this reduction is unknown [13], [14], [17]. Our data also suggest that the induction of negative regulators such as SIGGIR, TOLLIP and A-20 might be part of the regulatory process that dampens inflammatory enterocyte potential after birth.

As helpful as animal models for NEC are in understanding mechanisms of inflammation in the newborn premature gut, they do not mimic the actual condition of human NEC. Accordingly, we have established human intestinal models of gut development to determine their response to colonizing bacteria. In this study, we have used the *ex vivo* fetal intestinal xenograft model in addition to primary enterocytes isolated from freshly resected fetal and NEC small intestine to demonstrate that an immaturity in innate immune inflammatory response genes are developmentally regulated and undoubtedly important in explaining the excessive NEC inflammatory response. We have had extensive experience with the fetal human intestinal xenograft model [7]–[9], [15] and have used this *ex vivo* model system to provide functional relevance to *in vitro* cellular studies. In addition to the developmental upregulation of TLRs, signaling molecules and transcription factor genes, underexpression of negative regulators of TLR signaling appear to be the key to the excessive inflammatory response in immature enterocytes. Again this underexpression of negative regulators was further reduced in resected NEC intestine (**Figure 1C**). The significant role of specific negative regulators in reducing the IL-8 inflammatory response was confirmed by selective knockdown experiments using primary enterocytes isolated from fetal intestine (**Figure 5A**). These observations collectively support our hypothesis and may help to explain the role of enterocyte immaturity in the excessive inflammatory response to MAMPs in premature infants that may lead to NEC.

Using developing small intestinal xenografts as an *ex vivo* model to confirm cellular studies, we provide additional supporting evidence that an immaturity in innate immune inflammatory response genes accounts for the excessive inflammatory response to inflammatory stimuli (**Figure 2**). Using this model, we have previously reported that steroids can accelerate functional inflammatory maturation in the intestine [9]. In this study, our data show that part of the immune maturation is mediated by the reduction of TLR2 and TLR4 expression and induction of specific negative regulators of TLR signaling (**Figure 3**). This observation strongly suggests that an altered developmental regulation of immature inflammatory response genes with intrauterine cortisone use may be the key to the significant reduction of NEC in preterm infants.

In addition, to confirm the developmental expression of innate immune response genes has a functional significance, we determined protein levels of A-20 (**Figures 4C** **and** **5B**) as a selective confirmation that proteins as well as genes were affected in fetal/NEC enterocytes.

In conclusion, these observations in human fetal and resected NEC intestine suggest a mechanism for the age-related incidence of excessive inflammation in NEC prematures and a possible pathophysiologic basis for the role of prematurity in its expression. Furthermore, by establishing a developmental regulation of the innate immune inflammatory response in human fetal enterocytes as the basis for this disease, the use of known trophic factors (e.g. EGF, TGF-β and cortisone) at appropriate times during gestation or after delivery could result in a prevention strategy for the care of premature infants. However, when the use of cortisone was recommended, it was determined that early cortisone exposure, particularly in the postpartum period, had adverse consequences on other organ systems [24], [25]. Therefore, it becomes important to identify the appropriate steroid responsive period *in vitro* and the right dose to be used in multi-center trials before recommendations for routine clinical care can be made.

## Materials and Methods

### Chemicals

All the chemicals were of analytical grade obtained from the Sigma (St. Louis, MO). Reagents related to cell culture were obtained from GIBCO BRL (Rockville, MD). All reagents for ELISA and recombinant IL-1α and epidermal growth factor (EGF) were obtained from R&D Systems (Minneapolis, MN).

### Human intestinal models

#### Resected intestinal tissues

Resected ileal segments were collected after obtaining informed consent from Partners Human Research Committee (Protocol #1999-P-003833 [Walker]). Selected segments of resected ileum for necrotizing enterocolitis were obtained from multiple institutions Partners for Human Research Committee (Protocol #1999-P-003833 [Walker], Committee for Human Studies Instituto de Nutricion, Universidad de Chile [Uauy], and the Institutional Review Board University of Chicago (Protocol #13109B) [Claud]) and handled similarly. Each of the NEC specimens were obtained from prematures, weighing between 1200 and 1500 gms exhibiting pneumatosis intestinalis approximately two to three weeks after birth and after approximately one week of oral feedings. Marginal normal ileal tissue adjacent to the diseased intestine was obtained. Primary enterocytes were only isolated from freshly resected NEC tissue at MGH*f*C or Children's Hospital. Control tissues were obtained from children under six years of age who had elective small intestinal resections for intestinal anomalies (e.g., ileal atresia, annular pancreas, etc). Again marginal small intestine was taken from the operating room for primary enterocyte separation and LCM for RNA. Informed written consent was obtained from the parents/guardian for this study. In selected segments laser capture microdissection was used to obtain epithelial RNA.

#### Fetal tissue

Human small intestine was obtained from elective prostaglandin/saline-induced therapeutic abortion of 12 to 16 wk fetuses after informed written consent from Partners for Human Research Committee (Protocol 1999-P-003833 [Walker]). All experiments involving utilizing human tissues were approved by Partners for Human Research Committee (Protocol #1999-P-003833 and Committee for Human Studies Instituto de Nutricion, Universidad de Chile.

#### Small intestinal xenografts

Two cm of fetal ileum was implanted subcutaneously into SCID mice as described previously [6]–[8]. Successful human ileal xenografts were harvested at ten weeks posttransplantation and analyzed. Histology was performed and only specimens with normal histology were used in subsequent analysis. To obtain immature and mature human intestinal xenografts, the tissue was harvested at 20-week and 30-week post transplantation, respectively, challenged with inflammatory stimuli (LPS 50 ng/ml or IL-1β 1 ng/ml) and the intestinal IL-8 response was measured by ELISA as described previously [7], [9]. In selected experiments xenograft tissue were frozen at –80°C for RNA extraction as described previously [15] and few were subjected to laser capture microdissection (see below for details) for epithelial RNA isolation. A LDH and sucrase assay were performed to monitor the viability and to determine the mucosal integrity during the course of the experiments. All animal experiments were approved by the Subcommittee on Research Animal Care, Massachusetts General Hospital (Protocol #2005N000040).

### Epithelial Cell Isolation and Culture

The isolation and culture of primary enterocytes from resected child small intestine, fetal xenografts and resected NEC intestine has been reported previously [15], [26], [27]. Briefly, segments of small intestine were washed in sterile medium twice and incubated with a 1.25% trypsin–0.5 mmol/L ethylenediaminetetraacetic acid solution at room temperature before intestinal crypts were isolated by incubating with media containing collagenase type IV (200 U/mL). The dissociated epithelial cells were washed and resuspended in OptiMEM supplemented with 20 ng/mL EGF, 10 µg/mL insulin and 4% FBS before incubating at 32°C with 5% CO_2_. These primary enterocyte cultures were confirmed by immunological technique as described before [27] and used to assess the inflammatory response to various pro-inflammatory stimuli as described before [9], [10].

### Analytical Method

The IL-8 ELISA, protein determination, lactate dehydrogenase (LDH) cytotoxicity assay, immunofluorescence, total RNA isolation and quantitative real time qRT-PCR were described previously and detailed in previous publications [7], [9], [27].

### Laser capture microdissection (LCM) of epithelial RNA

The epithelial cells were isolated by LCM from fresh resected intestinal tissue or from immature (20-week) and mature (30-week) intestinal xenografts. The tissue was frozen in OCT^®^, then eight micron thin cryo-sections were cut and mounted on microscopy slides (Gold Seal Rite-On Micro Slides, Portsmouth, NH) and epithelial cells were isolated as described previously [26].

Briefly, thin intestinal cryosections were fixed in 70% ethanol, rinsed with RNAse free water and toluidine blue stained followed by dehydration in increasingly concentrated ethanol and xylene before laser capture microdissection of the epithelial cells (LCM). Approximately 600 toluidine blue stained epithelial cells from each section of the mucosa were captured onto polyethylene collecting caps (Macro Cap, Arcturus, MDS Analytical Technologies, Sunnyvale, CA) [26]. The total RNA was collected according to the Qiagen using their kit (Qiagen, CA) and the quality of RNA was assessed by agilent bioanalyzer (Waldbronn, GmbH) using RNA 6000 pico chip. Then the total RNA was used forqRT-PCR analysis for specific mRNA with GAPDH as a standard housekeeping gene for normalization in using SYBR green mastermix [26].

### SDS-PAGE and Western blot (immunoblot analysis)

In general, protein samples will be lysed and quantitated by the BCA method (Pierce) using BSA as a standard [27], [28]. SDS-PAGE and Western blot analysis will be completed as previously described in our laboratory [9], [10], [23]. Bands will be quantified by densitometry (NIH Image® prism).

### Knock down of TOLLIP expression by siRNA transfection

Specific siRNA oligonucleotide were designed and purchased from Invitrogen® (San Diego, CA). Epithelial cells at 50% confluence were transfected with 10 nM of specific siRNA or negative control using RNAiMAX kit according to manufacturer's recommendations. Transfection efficiency was assessed by transfecting the cell with BLOCK-iT™ Alexa Fluor Red fluorescent control and quantifying total number of cells by DAPI staining and transfected cells by red fluorescence to calculate the efficiency. Specific knockdown efficiency for each gene was measured by qRT-PCR and/or by western blots.

### Flow Cytometry

Cell surface expression of TL4 was determined by flow cytometric analysis. The expression n the immature (H4 cells) [8], [10] and mature enterocytes (CaCo-2 cells) [8], [10] was determined by staining for 1.10^6^ cells. Isolated cells were washed once with FACS media (BPS with 1% BAS, 01.% sodium azide) and blocked with FACS media containing 5% mouse serum for 30 min on ice. After washing, cell suspension were stained with PE-conjugated mouse anti-human TLR4 mAb (HTA 125, eBioscience) at 1 in 20 dilution for 30 min on ice in the dark, washed three times with FCS media and fixed in 1% paraformaldehyde. Negative controls were prepared by incubating the cells with PR-labeled isotope control mAb.

### FACS analysis

The cell surface expression of TLR4 expression was analyzed by FACS analysis. The H4 cells were used as a model for immature enterocytes and CaCo-2 cells were used as a model for mature enterocytes. One million H4 cells or Caco2 cells were re-suspended and washed once with FACS buffer (PBS containing 1% BSA and 0.1% NaN3) and blocked with FACS buffer containing 10% mouse serum for 30 min on ice. The cells were incubated with anti-TLR4 mouse mAb conjugated PE (Clone # HTA125, eBioscience, San Diego, CA) or IgG2a specific isotype control antibody (eBioscience, San Diego, CA) for 30 minutes at 4°C in the dark at the concentration recommended by the manufacturers. Then the cells were washed for three times with FACS buffer and then fixed with 1% Paraformaldehyde. Then the stained samples were analyzed on a Accuri C6 Flow Cytometer (BD Bioscience FACScan) with Cell Quest software. Dead cells and debris were excluded from analysis by gates set on forward and side angle light scatter. The results shown are histograms of flow cytometric analysis of H4 and Caco2 cells.

### Statistics

Results were presented as the mean ± SE. The effects of age and treatment on chemokine secretion were analyzed by a two-way ANOVA and with post-hoc two-tailed unpaired t test. Differences with a P value of <0.05 were considered significant.

## References

[1] Neu J, Walker WA (2011). Necrotizing enterocolitis.. N Engl J Med.

[2] Lin PW, Stoll BJ (2006). Necrotising enterocolitis.. Lancet.

[3] Grave GD, Nelson SA, Walker WA, Moss RL, Dvorak B (2007). New therapies and preventive approaches for necrotizing enterocolitis: report of a research planning workshop.. Pediatr Res.

[4] Henry MC, Moss RL (2009). Necrotizing enterocolitis.. Annu Rev Med.

[5] Claud EC, Walker WA (2001). Hypothesis: inappropriate colonization of the premature intestine can cause neonatal necrotizing enterocolitis.. FASEB J.

[6] Halac E, Halac J, Begue EF, Casanas JM, Indiveri DR (1990). Prenatal and postnatal corticosteroid therapy to prevent neonatal necrotizing enterocolitis: a controlled trial.. J Pediatr.

[7] Nanthakumar NN, Klopcic CE, Fernandez I, Walker WA (2003). Normal and glucocorticoid-induced development of the human small intestinal xenograft.. Am J Physiol Regul Integr Comp Physiol.

[8] Nanthakumar NN, Fusunyan RD, Sanderson I, Walker WA (2000). Inflammation in the developing human intestine: A possible pathophysiologic contribution to necrotizing enterocolitis.. Proc Natl Acad Sci USA.

[9] Nanthakumar NN, Young C, Ko JS, Meng D, Chen J (2005). Glucocorticoid responsiveness in developing human intestine: possible role in prevention of necrotizing enterocolitis.. Am J Physiol Gastrointest Liver.

[10] Claud EC, Lu L, Anton PM, Savidge T, Walker WA, Cherayil BJ (2004). Developmentally regulated IkappaB expression in intestinal epithelium and susceptibility to flagellin-induced inflammation.. Proc Natl Acad Sci.

[11] Ohnuma K, Yamochi T, Uchiyama M, Nishibashi K, Iwata S (2005). CD26 mediates dissociation of TOLLIP and IRAK-1 from caveolin-1 and induces upregulation of CD86 on antigen-presenting cells.. Mol Cell Biol.

[12] Xiao H, Gulen MF, Qin J, Yao J, Bulek K (2007). The Toll-interleukin-1 receptor member SIGIRR regulates colonic epithelial homeostasis, inflammation, and tumorigenesis.. Immunity.

[13] Otte JM, Cario E, Podolsky DK (2004). Mechanisms of cross hyporesponsiveness to toll-like receptor bacterial ligands in intestinal epithelial cells.. Gastroenterology.

[14] Melmed G, Thomas LS, Lee N, Tesfay SY, Lukasek K (2003). Human intestinal epithelial cells are broadly unresponsive to Toll-like receptor 2-dependent bacterial ligands: implications for host-microbial interactions in the gut.. J Immunol.

[15] Lu L, Bao Y, Khan A, Goldstein AM, Newburg DS (2008). Hydrocortisone modulates cholera toxin endocytosis by regulating immature enterocyte plasma membrane phospholipids.. Gastroenterology.

[16] Fusunyan RD, Nanthakumar NN, Baldeon ME, Walker WA (2001). Evidence for an innate immune response in the immature human intestine: toll-like receptors on fetal enterocytes.. Pediatr Res.

[17] Abreu MT (2010). The ying and yang of bacterial signaling in necrotizing enterocolitis.. Gastroenterology.

[18] Sodhi CP, Sh X-H, Ricardson WM, Grant ZS, Shapiro RA (2010). Toll-like receptor-4 inhibits enterocyte proliferation via impaired β-catenin signaling in necrotizing enterocolitis.. Gastroenterology.

[19] Lotz M, Gütle D, Walther S, Ménard S, Bogdan C (2006). Postnatal acquisition of endotoxin tolerance in intestinal epithelial cells.. J Exp Med.

[20] Harada K, Isse K, Sato Y, Ozaki S, Nakanuma Y (2006). Endotoxin tolerance in human intrahepatic biliary epithelial cells is induced by upregulation of IRAK-M.. Liver Int.

[21] Stathopoulos A, Levine M (2002). Dorsal gradient networks in the Drosophila embryo.. Dev Biol.

[22] Jilling T, Simon D, Lu J, Meng FJ, Li D (2006). The roles of bacteria and TLR4 in rat and murine models of necrotizing enterocolitis.. J Immunol.

[23] Caplan MS, Simon D, Jilling T (2005). The role of PAF, TLR, and the inflammatory response in neonatal necrotizing enterocolitis.. Semin Pediatr Surg.

[24] Hayes EJ, Paul DA, Stahl GE, Seibel-Seamon J, Dysart K (2008). Effect of antenatal corticosteroids on survival for neonates born at 23 weeks of gestation.. Obstet Gynecol.

[25] Doyle L, Davis P (2000). Postnatal corticosteroids in preterm infants:systematic review of effects on mortality and motor function, J Paediatr.. Child Health.

[26] Weng M, Walker WA, Sanderson IR (2007). Butyrate regulates the expression of pathogen-triggered IL-8 in intestinal epithelia.. Pediatr Res.

[27] Lu L, Khan A, Walker WA (2009). ADP-ribosylation factors regulate the development of CT-signaling in immature human enterocytes. AJP: Gastrointestinal and Liver Physiology 296(6):G1221-G1229.. PMID.

[28] Lowry OH, Rosebrough NJ, Farr AL, Randall RJ (1951). Protein measurement with the Folin phenol reagent.. J Biol Chem 1951;.

